# Use of Creative Frameworks in Health Care to Solve Data and Information Problems: Scoping Review

**DOI:** 10.2196/55182

**Published:** 2024-09-13

**Authors:** Elisabeth Veronica Mess, Frank Kramer, Julia Krumme, Nico Kanelakis, Alexandra Teynor

**Affiliations:** 1 Institute for Agile Software Development Technical University of Applied Sciences Augsburg Augsburg Germany; 2 Chair of IT Infrastructure for Translational Medical Research University of Augsburg Augsburg Germany; 3 Technical University of Applied Sciences Augsburg Augsburg Germany

**Keywords:** creative frameworks, data and information problems, data collection, data processing, data provision, health care, information visualization, interdisciplinary teams, user-centered design, user-centered data design, user-centric development

## Abstract

**Background:**

Digitization is vital for data management, especially in health care. However, problems still hinder health care stakeholders in their daily work while collecting, processing, and providing health data or information. Data are missing, incorrect, cannot be collected, or information is inadequately presented. These problems can be seen as data or information problems. A proven way to elicit requirements for (software) systems is by using creative frameworks (eg, user-centered design, design thinking, lean UX [user experience], or service design) or creative methods (eg, mind mapping, storyboarding, 6 thinking hats, or interaction room). However, to what extent they are used to solve data or information-related problems in health care is unclear.

**Objective:**

The primary objective of this scoping review is to investigate the use of creative frameworks in addressing data and information problems in health care.

**Methods:**

Following JBI guidelines and the PRISMA-ScR framework, this paper analyzes selected papers, answering whether creative frameworks addressed health care data or information problems. Focusing on data problems (elicitation or collection, processing) and information problems (provision or visualization), the review examined German and English papers published between 2018 and 2022 using keywords related to “data,” “design,” and “user-centered.” The database SCOPUS was used.

**Results:**

Of the 898 query results, only 23 papers described a data or information problem and a creative method to solve it. These were included in the follow-up analysis and divided into different problem categories: data collection (n=7), data processing (n=1), information visualization (n=11), and mixed problems meaning data and information problem present (n=4). The analysis showed that most identified problems fall into the information visualization category. This could indicate that creative frameworks are particularly suitable for solving information or visualization problems and less for other, more abstract areas such as data problems. The results also showed that most researchers applied a creative framework after they knew what specific (data or information) problem they had (n=21). Only a minority chose a creative framework to identify a problem and realize it was a data or information problem (n=2). In response to these findings, the paper discusses the need for a new approach that addresses health care data and information challenges by promoting collaboration, iterative feedback, and user-centered development.

**Conclusions:**

Although the potential of creative frameworks is undisputed, applying these in solving data and information problems is a minority. To harness this potential, a suitable method needs to be developed to support health care system stakeholders. This method could be the User-Centered Data Approach.

## Introduction

### Background

Despite advancing digitization in the health care sector, problems still hinder health care stakeholders in their daily work while collecting, processing, and providing health data or information. These problems can be seen as data or information problems.

This scoping review aims to review if creative frameworks were used to solve data or information problems in health care.

A secondary objective was to understand how these creative frameworks were applied in detail to understand their possible impact on solving data and information problems.

### Definition of Data and Information

**Figure 1:**
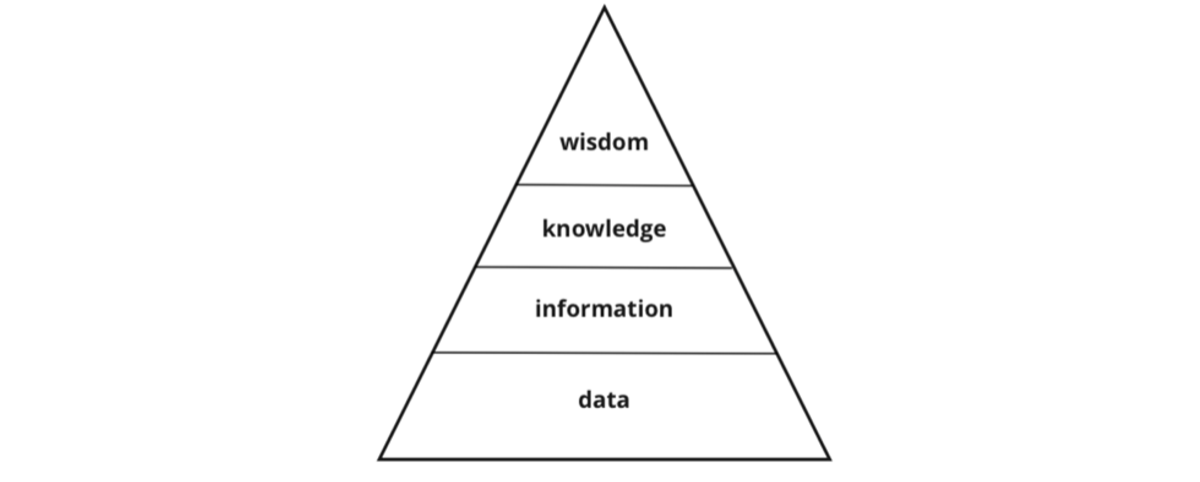
Data Information Knowledge and Wisdom Hierarchy (DIKW).

The correlation between data and information can be visualized with the information pyramid [1], whereas data are necessary to get information that leads to further possible knowledge and wisdom (Figure 1).

Data are defined as “facts and statistics collected for reference or analysis [and] the quantities, characters, or symbols on which a computer performs operations” [2]. Data gain value when analyzed and structured into information, which is crucial for extracting insights and making informed decisions. The word information is defined by [3] as “notice, news or advice communicated by word or writing*.*” Claude Shannon, the father of modern information theory, quantifies information as a measure of uncertainty or surprise in a message, introducing the concept of entropy as in [4].

Over the past decades, replacing “information” with “data” has become common. The word data now includes entities and meaning [5]. However, this can cause misunderstandings, so it is important to consider the specific discipline’s framework [6].

In general, data can be categorized as structured, unstructured, or semistructured. Structured data are organized and formatted in a predefined manner, with each element assigned a specific type, often found in databases or spreadsheets (eg, CSV and XML) [7]. In health care, this type of data includes electronic health records (patient demographic information, diagnosis codes, and medication lists) [8].

Unstructured data lacks a predefined structure, including text, images, audio, video, and social media posts, making it harder to process automatically [9,10]. In health care, this data can be descriptions or medical history, mainly text in free written form. Semistructured data combine elements of both, with some organizational structure but not a rigid schema (eg, JSON files, XML documents with optional tags, or a combination of checkboxes and descriptions).

### The Importance of Data and Information in Health Care

Medical decision-making is one area that benefits crucially from leveraging data and information. Health care providers can gain valuable insights into patients’ diseases or treatment outcomes [11]. Also, it can improve the patient’s health status or satisfaction (cf. [12,13]). It can also significantly influence the quality of patient care and patient safety. By continuously analyzing, for example, clinical data and patient feedback, it is possible to identify areas needing improvement [14]. Another aspect is positively adjusting operational activities by analyzing patient flow, operational processes, and workflows [15].

### Data and Information Problems in Health Care

Despite the importance of data and information, health care professionals and practitioners must often make highly calculated and accurate decisions with limited information, resources, and knowledge [16-20]. That puts a more significant burden on the staff and prevents them from harnessing the potential value of data and information. These problems can be seen as data and information problems.

A data problem is present when the necessary data for further processing, analysis, and provision are unavailable or cannot be collected (sufficiently). That can involve anomalies, discrepancies, or limitations within the data itself or difficulties related to data processing, integration, quality assurance, or ethical considerations.

An information problem can arise when the information is not provided sufficiently or is unrepresentative. This points to a challenge when seeking, evaluating, synthesizing, or using information sources and can encompass difficulties related to information scarcity, retrieval, relevance assessment, credibility assessment, information organization, or ethical considerations.

It is important to stress that (regardless of these definitions) the complexity of these problems constantly changes depending on the context and the target group. Due to that, a “one-fits-all” concept or solution is unrealistic.

### Creative Frameworks in Software Development in Health Care

Diverse approaches are used in various fields to stimulate innovative thinking, generate novel ideas, and foster problem-solving. Some of these well-known creative approaches are design thinking, user-centered design (UCD) or human-centered design (HCD), lean UX [user experience], lean startup, and service design. These creative approaches are frameworks that provide a structured approach or guidelines that define the overall context, principles, and objectives for fostering creativity. They often offer an overarching perspective and may encompass multiple creative methods.

In contrast, an individual creative method refers to a specific technique or tool. They are typically more focused compared to frameworks. Creative methods include mind mapping, storyboarding, 6 thinking hats, and scamper or interaction room.

In the context of health care, the approaches design thinking, UCD, and HCD are often successfully used to develop user-centered software or hardware to support health care professionals’ daily practice [21-24]. This may be due in no small part to the fact that specifically human-centered design is anchored in ISO (International Organization for Standardization) standards, which aim to ensure consistency, quality, safety, efficiency, and interoperability in different disciplines.

An example is ISO 9241, focusing on the Ergonomics of human-system interaction. In the ISO 9241:2019, Part 210 focuses on a human-centered design for interactive systems. The HCD process is divided into different activities and sub-activities and focuses on their responsibilities for human-centered quality in software development [25].

### Creative Frameworks and Data or Information Problems in Health Care

Data and information problems can vary significantly in their complexity—from anomalies or limitations in the data to information overload. Depending on the context, further complex combinations of problems can arise and pose challenges for all relevant target groups.

Many of these problems cannot be solved by one discipline alone. Interdisciplinary and human-centered exchange is necessary to find the best possible solution.

Creative frameworks are ideal here; they provide a structure and help find creative, human-centered solutions in an interdisciplinary team.

An initial keyword search [26] conducted by the authors showed that data and information problems in health care were partially solved by using creative frameworks. A total of 100 papers were screened for two acceptance criteria: (1) data or information problem present and (2) a creative framework used to solve it. The screening resulted in 4 papers that met the criteria. However, these results were not representative (small sampling size, incomplete data), so it was impossible to deduce the extent to which creative frameworks are disseminated and applied in detail in the context of data and information problems and whether this output is just a coincidence. After all, no other publication was found that deals with this focus, which led to conducting a scoping review to obtain further information.

## Methods

### Overview

In this work, a scoping review was conducted to determine if creative frameworks were used to solve data or information problems in health care. A secondary objective was to understand how creative frameworks were applied in detail to understand their possible impact on solving data and information problems.

**Figure 2:**
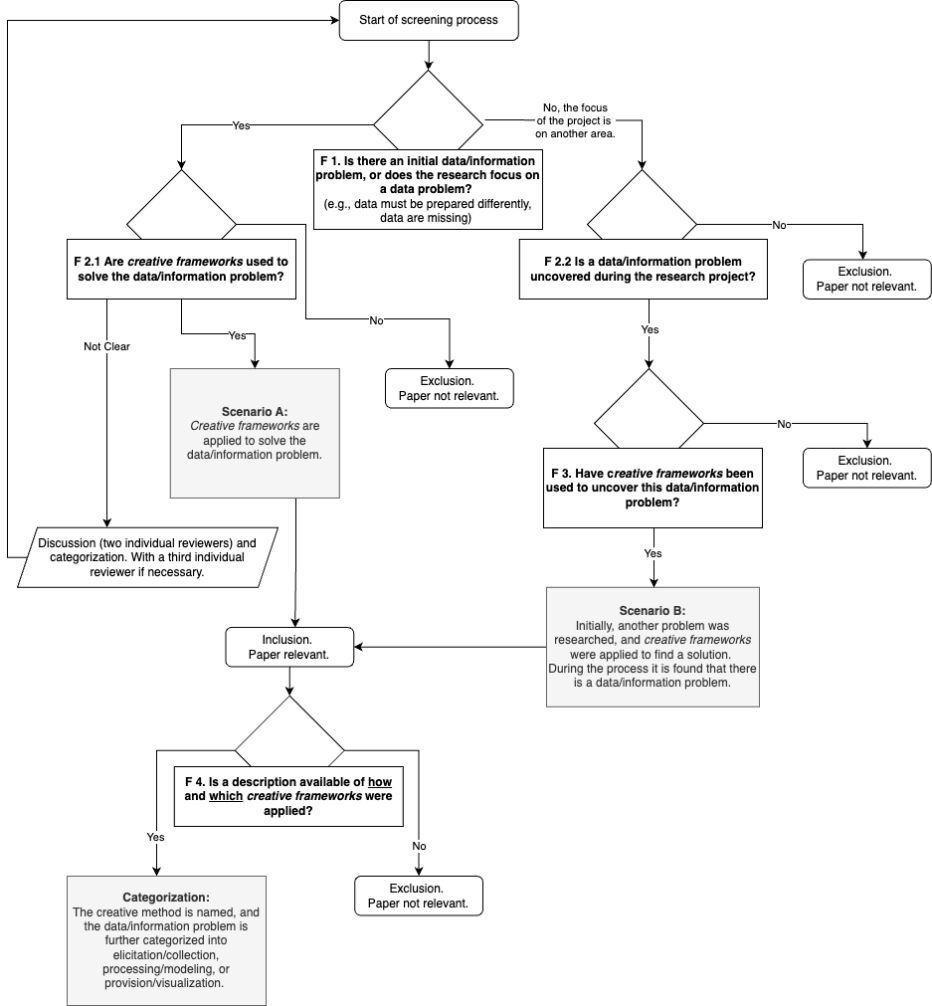
Overview of the decision flow diagram with the different scenarios.

**Figure 3:**
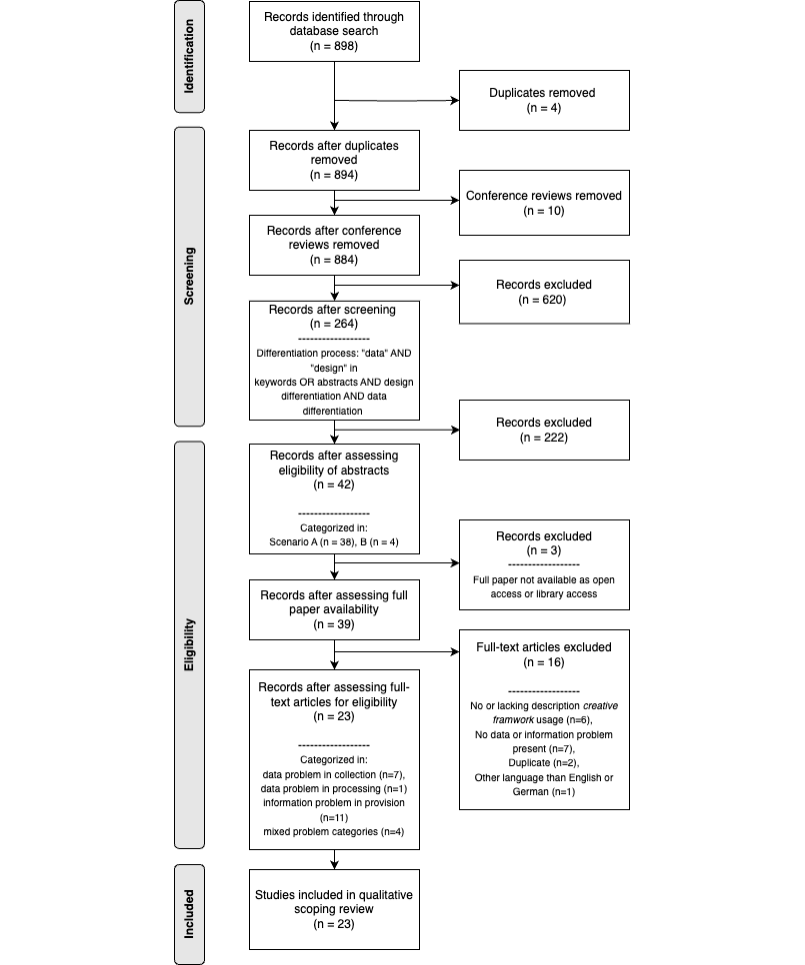
PRISMA-ScR (Preferred Reporting Items for Systematic Reviews and Meta-Analyses extension for Scoping Reviews) flow diagram, including the different rounds and scenario decisions conducted.

The scoping review was conducted using the guidelines of the Joanna Briggs Institute (JBI) [27] and the PRISMA-ScR (Preferred Reporting Items for Systematic Analyses and Meta-Analyses extension for Scoping Reviews); see Figures 2 and 3. It provides researchers with a preferred reporting item and a checklist, as in [28]. Papers meeting the criteria for inclusion were chosen with a developed decision flow diagram (see Figure 2).

### Research Question

The following research question was explored: Have creative frameworks been used in solving “data or information problems” in medicine or health care, for example, the collection, processing, or provision of data?

### Search Strategy

SCOPUS, ACM, and IEEE libraries were initially considered suitable because they are known for their extensive coverage of academic literature (journals, conference proceedings, reports), particularly in computer science. Additionally, the published papers are peer reviewed, and they provide advanced search features.

During initial keyword testing, it was found that the libraries ACM and IEEE are unsuitable for this scoping review as they have either keyword or content group limitations. Due to the number of different keyword spellings (see Tables I and II) and the need for a parallel search in the document title, abstract, and keywords, SCOPUS was chosen.

Studies published in German and English from 2018 to 2022 were included. The search was performed on July 6, 2022.

### Search String and Source Selection

The main search strings were »data« AND »design« in the categories of Computer Science, Medicine, Nursing, and Health Professions. Due to the more common definition of data, which refers to a data-based definition of information as mentioned in the Introduction, the keyword information was not included. However, further analysis of the query results will interpret the word data and distinguish between data and information.

Concerning the selection of creative frameworks*,* the authors decided to focus on UCD, HCD, and design thinking because they have already proven successful in software development. Additional keywords like service design, lean UX, and lean startup were tested for relevance. Service design returned a significant query result with approximately 200 papers; thus, it was included in the search string. Lean UX and lean startup delivered only a handful, and the keywords were referenced neither in the title nor abstract nor keywords, so these 2 creative frameworks were excluded from the search string.

The remaining 4 keywords for creative frameworks were searched using the title, abstracts, and authors’ keywords. As these main keywords are insufficient to differentiate when and how creative frameworks have been used (collection, processing, provision or visualization), further subkeywords were defined. That was primarily due to different spelling and general writing options for UCD and HCD, as seen in Multimedia Appendix 1. The overall number of keywords and substrings was 32 (including main and differentiation keywords). Definitions of the keywords are listed in a separate appendix.

All retrieved papers were matched against a decision flow diagram (see Figure 2). This step was included to guarantee that all perspectives and approaches to using creative frameworks in data and information problem-solving were included. Therefore, the papers were further categorized in this diagram with different implementation scenarios of creative frameworks (creative frameworks used yes/no, data or information problem solving with creative frameworks yes/no).

### Study or Source of Evidence Selection

All identified papers were collected within an EndNote database and shared with all reviewers after the search. The differentiation of the paper focused on screening the document title, abstract, and keywords. This step was conducted individually with smart groups by each reviewer. In EndNote, smart groups can be populated automatically by predetermined criteria. It can be described as “Search in this group in the title, abstract, keywords for keywords X, Y, Z.”

Duplicates and conference reviews were removed within the screening process.

Potentially relevant sources were retrieved in full while checking the eligibility. Any disagreements between the reviewers at each stage of the selection process were resolved through discussion or with an additional reviewer.

The search results and study inclusion process are presented in the PRISMA-ScR flow diagram, as shown in Figure 3.

## Results

### Overview

The literature search retrieved 898 items from SCOPUS. After the removal of duplicates (n=4) and conference reviews (n=10), titles, abstracts, and keywords of 884 papers were screened according to the inclusion and exclusion criteria (description scenarios see Table 1 and decision flow diagram see Figure 2).

The overall process can be viewed with the PRISMA ScR flow diagram (see Figure 3). Afterward, further research results are presented.

**Table 1:** Description of 2 defined scenarios of the decision flow diagram.

Scenarios	Descriptions
Scenario A	Creative frameworks are applied directly to solve a data or information problem. A data problem was evident at the beginning of the study, and a creative method was used to solve a data or information problem. That means the awareness of the data problem was high, and choosing creative frameworks to solve the problem was a conscious decision.
Scenario B	Creative frameworks are applied indirectly to solve a data or information problem. That means the awareness of a data or information problem came later in the research process, and a creative method was conducted first.

### Findings

A total of 42 abstracts and articles were assessed for eligibility, and 39 papers could be retrieved as full texts. Two reviewers screened the remaining 39 full papers individually and categorized them into data problems (elicitation or collection, processing), information problems (provision/visualization), and mixed problems (data and information problems).

During this process, 23 papers were included (see Table 2), and 16 were excluded (see Table 3). Any disagreements between the 2 reviewers were discussed, and a third reviewer was consulted in cases of doubt. A detailed overview of the categorization of all 39 papers can be found in Multimedia Appendix 2.

**Table 2:** Summary of available full-text papers included.

Problem category	Number of papers
Data elicitation or collection	7
Data processing or modeling	1
Data, information provision, or visualization	11
Mixed problems (data and information problem present)	4
Total amount of papers included	23

**Table 3:** Summary of available full-text papers excluded.

Duplicate	2
Other languages used than English or German	1
No data or information problems described	7
Insufficient or missing description of the creative framework	6
Total amount of papers excluded	16

#### Key Finding 1—General Minority

At the beginning of the scoping review, 898 papers were identified. In the end, 23 papers met all inclusion criteria. With these papers, the research question, “Have creative frameworks (UCD, HCD, design thinking, or service design) been used in solving ‘data/information problems’, for example, in the collection, processing, and/or provision of data?” can be answered with yes but it is a minority. Only 23 papers used creative frameworks to solve data and information problems in health care.

At this point, the question arises as to why. Is the application of creative frameworks in this context insufficient, or was the focus on solving specific data and information problems with creative frameworks not given? Or are there other factors contributing to this, like the lack of insufficient description and exclusion (see Key Finding 2)? Since creative frameworks have proven successful in developing health care software and hardware in the health care sector, transferring these frameworks and techniques to solve data and information problems does not seem so far-fetched.

#### Key Finding 2—Lacking Descriptions of Problem Statements and Method Description

From all query results, 264 had the keywords »data« AND »design« AND 1 design differentiation (e.g., design thinking) AND 1 data differentiation (e.g., data collection).

To better understand what, how, and why the creative frameworks were applied, the papers (n=264) were further screened for 1. data/information problem present and 2. Creative framework used. In the first step of this activity, the reviewers focused on the title, abstract, and keywords and searched for a problem statement and the mention of a creative framework (that goes beyond mentioning it in the keywords). If a problem was mentioned, it was checked to see whether it was a data or information problem.

It is important to stress that the word “problem” does not mean that every paper needs to focus on a major or new problem and try to find a solution. In some cases, the problem may be rather “small” and very specific, for example, the UI design no longer works for the target group and needs to be adapted. In other cases, the problem is more general and indicates, for example, a lack of sufficient data for further research. In total, 222 papers had to be excluded because they did not contain a problem statement that referred to a data or information problem or because no creative framework was mentioned. In these cases, it is difficult to completely understand the authors’ thoughts and decision-making process. Further clarification with the authors could help, but it is beyond the scope of this review.

From the remaining 39 papers that were checked for eligibility (full paper read), only 23 could be included (see Multimedia Appendix 3 for further details). In many contributions, the data or information problem was not described in detail (n=7) and only briefly mentioned in the abstract. The description of how creative frameworks were applied was insufficient or missing in 6 papers. That could point to the fact that the authors did not feel a more detailed description was necessary or that they maybe had difficulty applying the creative framework. Further details are provided in Multimedia Appendix 4 showing 6 examples of included (n=2) and excluded (n=3) papers.

#### Key Finding 3—Initial Clarity About Problems Needed

During the screening and development of the decision framework for inclusion criteria, it was found that there might be different types of awareness when and how a creative framework is used to solve a problem. Either “We have a data or information problem, and we use creative frameworks to solve it” or “We have a problem, and we use creative frameworks to solve the problem or we use it to find out more about it. As a result, we found a data or information problem.”

Most papers (n=21) identified a data or information problem first (see Scenario A) and later decided to apply creative framework to solve it. In only 2 cases (n=2) did the researchers address a different type of problem and identified a data or information problem by applying a creative framework (referring to Scenario B). This might point to the fact that the problem must be apparent first before deciding to use a creative framework to solve it.

#### Key Finding 4—Creative Frameworks Most Applied in Case of Information Problems

Most of the included papers (n=11) dealt with an information problem, and 8 dealt with a data problem. Some papers (n=4) dealt with a combination of data and information problems and were categorized as mixed problems (problem in data collection, data processing and information provision n=1, problem in data collection and data processing n=2, and problem in data collection and data provision n=1).

That could indicate that creative frameworks are particularly suitable for solving provision or visualization problems and less for other, more abstract areas such as data problems. Also, combining several creative frameworks to solve a data/information problem leads to a preferred visual (data) output.

However, the remaining 8 papers show that it is possible to use a creative frameworks for a more abstract problem. This could mean that creative frameworks have not yet been identified as suitable.

#### Key Finding 5—UCD is Most Frequently Used as a Creative Method

In the process of testing keywords for relevance, UCD, HCD, and design thinking were considered the most relevant as they have already proven to be successful in software and hardware development. After testing additional keywords such as service design, lean UX, and lean startup for relevance, only service design yielded a significant number of query results, approximately 200 papers. Therefore, it was incorporated into the search string. However, lean UX and lean startup produced only a handful of results, and these keywords were not referenced in the papers' title, abstract, or keywords. Consequently, these 2 creative frameworks were excluded from the search string.

Most of the final papers used only UCD to solve the problem at hand (n=16); the other remaining papers used either design thinking (n=2), HCD (n=1), other creative frameworks such as Rapid Contextual Framework (n=1), Design Science (n=1), or Design Study Methodology (n=2). Only a few papers used more than one creative method (n=2). That indicates that UCD is, compared to design thinking, HCD, or service design, the most common, best-known, or best-understood method to apply in health care settings.

Concerning the papers that used UCD (n=16), they can be divided into data collection problem (n=5), data processing problem (n=1), information problem (n=7), mixed data, and information problem (n=2).

This indicates that UCD is specifically used and probably suited for data collection problems and information problems. It is unclear if it is less suitable for data processing problems, as we do not know if it could be successfully applied or if researchers might know of the possibility of doing so.

#### Key Finding 6—no Unified Spelling

As shown at the beginning of Tables I and II (in Multimedia Appendix 1), many keywords (n=32) were used for this qualitative scoping review. Different spellings had to be considered, especially for the keyword »user-centered design« and further differentiations. That prolonged the overall process of the scoping review as it had to be continuously checked that all differentiations were included during the screening and eligibility process. Overall, this made it challenging to proceed efficiently and effectively. It is still possible that relevant papers might not be part of this work because not all relevant keywords and their spelling variants have been included.

### Closing the Gap: A New Approach Is Needed

The results of the initial literature research and scoping review demonstrate that creative frameworks have been used to address challenges in health care, such as information overload or data collection hindrances. However, this specific approach tends to be a minority.

Having identified an application gap, we propose a new idea—a Human-Centered Data Approach. With this, we aim to propose a structured way to minimize data and information problems in the health care setting.

The main goal is to analyze and integrate the users’ data and information needs and include these already at the earliest stage of development possible. This could influence the quality and quantity of data and the user's overall value.

First, because the user's awareness of data and information increases: “What are data or information, what importance do they have for me, and what information do I need?” Second, it is more likely that the applicability and understandability of the data and information for the user will increase.

It could also be possible to categorize data quality, quantity, or value according to stakeholders. A guideline could be part of the new approach to optimizing the data and, hence, information and include the user throughout the development process, for example, concepts or solutions.

Furthermore, developing this new approach within an interdisciplinary team is also essential. Different perspectives are needed to develop this new approach and conduct it correctly. Furthermore, to interpret potentials and risks that come with user-centered data (from the perspective of ethics: with more data value to the user, a loss could have even more severe (personal) consequences). Therefore, computer science, design, ethics, society stakeholders, and policy disciplines would be mandatory.

A sound basis for the approach could be to use an already existing and successful method, like HCD, UCD, or design thinking, and apply it to the context of data and information problems. That shall be done with an interdisciplinary team. The presentation of this will be part of the following publication.

## Discussion

Overall, this scoping review aimed to review if creative frameworks were used to solve data or information problems in health care. A secondary objective was to understand how these creative frameworks were applied in detail to understand their possible impact on solving data and information problems.

Identifying a relatively small number of papers (23 out of 898) that meet the inclusion criteria raises intriguing questions about applying creative frameworks in health care problem-solving. This small number may be due to the combination of data/information problems and creative frameworks, but this might also be because many papers had to be excluded due to a lack of clear data-/information problem description and method application (n=222), which were inclusion criteria. Authors may need to recognize the significance of providing detailed accounts of problem definitions and applying creative techniques to facilitate knowledge dissemination and the reproducibility of research outcomes.

Nevertheless, while it is established that methods using creative methods such as UCD, HCD, or design thinking have been successful in health care software and hardware development, their limited use in addressing data and information problems suggests that there may be barriers or unexplored opportunities. That prompts us to consider whether health care has yet to fully harness creative frameworks' potential in data-related challenges.

Most papers dealing with information problems (11 out of 23) hint at the suitability of creative frameworks for solving information provision and visualization challenges. This observation suggests that creative frameworks might be particularly suitable for addressing issues that require a visual representation of data. However, based on the findings, it is impossible to conclude that one specific creative framework is best suited for a specific problem. To do so, a higher number of results is needed.

Despite this, the presence of papers that tackle more abstract data problems (8 out of 23) shows that creative frameworks are not confined to information provision but can also be applied effectively to other complex data-related issues. This contrariness highlights the need for a more comprehensive understanding of when and how to apply creative frameworks across various health care contexts.

The differentiation between scenarios where researchers identify a data/information problem first or use creative frameworks to uncover such problems underscores the importance of problem clarity in adopting creative frameworks. Most cases (21 out of 23) had clarity about their problem statement in the beginning. However, cases where creative frameworks reveal hidden data or information problems (2 out of 23), indicate that creative frameworks can also be employed as exploratory tools to uncover latent issues. This nuanced perspective emphasizes the versatility of creative frameworks in health care research.

UCD, as the most used creative method (in 16 out of 23 papers), may suggest that it is the most familiar and widely accepted approach in health care settings. Its preference might also be attributed to its established reputation and applicability in health care contexts due to its references to ISO standards.

Another issue was the inconsistent spelling and terminology of UCD and HCD in the literature, which raises a practical concern for researchers conducting qualitative scoping reviews. The diversity in keywords and spellings could hinder the retrieval of relevant papers and add complexity to the literature search process. That emphasizes the importance of standardization in terminology and the need for researchers to employ comprehensive search strategies encompassing multiple spellings and variations to examine the available literature thoroughly.

With this work, we wanted to research if creative frameworks have been used to solve data or information problems in health care. We believe we have found an answer to this: yes, but it is a minority. We also wanted to highlight the need for a new approach to use the potential of creative frameworks to solve these problems. Additionally, we want to highlight that interdisciplinary discussion and evaluation, which includes the target group(s) in the earliest and all steps, is necessary to gain valuable data and information.

### Limitations

Concerning the scoping review, the selection of full papers for inclusion in this study depends on the authors' ability to provide a clear problem statement and a precise presentation of their methodology. It is possible that some of the excluded papers may have conducted research that could align with the acceptance criteria but failed to meet these criteria during the screening process due to their lack of description.

Furthermore, studies with significant findings are more likely to be published, while those with null or negative results may not be accessible, leading to an incomplete picture. Also, studies that SCOPUS does not index are not part of this review but can still hold valuable results.

Additionally, it is important to stress that selecting studies and extracting data in scoping reviews involves some subjectivity. Researchers must make judgment calls about study inclusion and data extraction, which can introduce bias and influence the overall outcome.

In fast-evolving fields like computer science, it is possible that this research does not cover all the relevant research despite the vast time span, as the overall screening process takes a longer time and is resource-intensive.

Lastly, the review heavily depends on available literature. Three articles could not be screened for eligibility because they were neither available as open access nor through online academic libraries.

### Conclusions

In conclusion, this scoping review offers valuable insights into using creative frameworks in health care research. The findings underscore the potential benefits of applying creative frameworks to address data and information problems while highlighting the need for greater clarity in problem definition, a more comprehensive exploration of creative methodologies, and improved reporting practices.

As health care evolves, embracing creative problem-solving approaches may prove instrumental in tackling complex data and information challenges.

We believe that the adaption or further development of UCD to the context of data and information problems could positively influence tackling data and information challenges. Ultimately, a new approach could initiate interdisciplinary dialogue, allowing researchers, practitioners, and stakeholders to reshape health care data management and improve patient care, informed decision-making, and the overall efficiency of health care systems.

Nevertheless, at this point, it is yet unclear if this will succeed or not. However, it could help to foster interdisciplinary discussion at the earliest development stage of software or hardware solutions.

## Appendix

Multimedia Appendix 1 Overview of 12 keywords for data and 17 keywords for design (primarily due to different notations).

Multimedia Appendix 2 Six search keywords are defined and described in detail.

Multimedia Appendix 3 Overview of all eligible papers (n=39).

Multimedia Appendix 4 Detailed examples (n=6) of included and excluded papers.

Multimedia Appendix 5 PRISMA-ScR checklist.

## Abbreviations

HCD: human-centered design
ISO: International Organization for Standardization
JBI: Joanna Briggs Institute
PRISMA-ScR: Preferred Reporting Items for Systematic Reviews and Meta-Analyses extension for Scoping Reviews
UCD: user-centered design
UX: user experience
