# Supplementary material for: Use of Creative Frameworks in Health Care to Solve Data and Information Problems: Scoping Review
Source: JMIR Hum Factors. 2024 Sep 13;11:e55182. doi: 10.2196/55182 (PMC11437220; doi:10.2196/55182)
Supplement: Multimedia Appendix 1 [file humanfactors_v11i1e55182_app1.docx]

**Table S1.** Overview of a total of 12 keywords for data.

| **Data** | Differentiation keywords data | | |
| --- | --- | --- | --- |
|  | Substring | | |
|  | data elicitation OR | data collection OR |  |
|  | data processing OR | data modeling OR |  |
|  | data provision OR | data visualization OR | data visualization OR |
|  | patient relevant data OR | patient data OR | medical data OR |
|  | personal data OR | private data |  |

**Table S2.** Overview of a total of 17 keywords for design, primarily due to different notations.

| **Design** | Differentiation keywords design | | |
| --- | --- | --- | --- |
|  | Substring | | |
|  | user-centred OR | user-centered OR |  |
|  | user-centred OR | user-centered OR |  |
|  | human-centred OR | human-centered OR |  |
|  | design thinking OR |  |  |
|  | service design OR |  |  |
|  | data design OR |  |  |
|  | user-centred data OR | user-centered data OR |  |
|  | user-centred data OR | user-centered data OR |  |
|  | Human-centred OR | human-centered OR |  |
|  | human-centred data OR | human-centered data OR |  |
