# Supplementary material for: Use of Creative Frameworks in Health Care to Solve Data and Information Problems: Scoping Review
Source: JMIR Hum Factors. 2024 Sep 13;11:e55182. doi: 10.2196/55182 (PMC11437220; doi:10.2196/55182)
Supplement: Multimedia Appendix 2 [file humanfactors_v11i1e55182_app2.docx]

### **Multimedia Appendix 2**

In the following, six search keywords are defined and described in further detail.

#### Keyword Definitions

Of all 32 relevant search keywords were six further described to provide an understanding of these terms by the authors.

| User-Centered Design: | “[…] is a software design methodology for designers and developers. […] it helps [to] make applications that meet the needs of their users. […].“ as in [1]. It is important to note that even though UCD emerged from HCI, all users are humans, but not all humans will be users of a specific product. The user-centered design process includes four iterative phases: 1) Specify context of use, 2) specify requirements, 3) produce design solutions, and 4) evaluation of design as mentioned in [2] or even simpler: Analysis, Implementation, Testing, and Evaluation. Users can be involved with certain techniques: „[…] Interviews and questionnaires […], Focus groups […], On-site observation […], walkthroughs […], Usability testing […]“ as in [3]. As described in [4], “[…] UCD methods are modular or identifiable processes involved in UCD practice. You should NOT think of UCD as merely usability testing or software engineering.” |
| --- | --- |
| Design Thinking: | “[…] design thinking […] describe themselves as being grounded in […] [the principles of Human-Centred Design, Creativity, Problem-Solving and Participation.] as in [5]. It aims to find new solutions for problems; it focuses consequently on the users, follows a structured and iterative process, and is usually conducted within an interdisciplinary team as in [6].  Service Design: the development of customer-oriented strategies and thus at the link of creative design and profitability as shown in [7]. A core for Service Design is the service, which has three primary spheres: care, response, and access, as described in [8]. |
| Service Design: | “[…] is a multidisciplinary approach to designing and improving services that focuses on creating meaningful and valuable experiences for users, customers, and stakeholders. It involves understanding user needs, mapping service processes, and designing service touchpoints to enhance the quality, efficiency, and user satisfaction of a service. Service design typically integrates principles from fields such as design thinking, user experience (UX) design, and systems thinking to create holistic and user-centered service experiences. […].” [9] |
| Data Elicitation: | “The word ‘elicitation’ encompasses various methods of data collection, which may differ depending on the discipline. For example, in the field of computer science (software and hardware development) and design (graphic design, UI/UX design), expert or semi-structured interviews, field observations, and contextual surveys or usability tests, among others, are essential for collecting various data.”  Data Processing: “[…] The work that is done on data is called data processing. This processing could be anything like addition, subtraction, or comparison. It is this processing that converts data into information. The computer processes data to give information. To get information from computer – Data are input into the computer, computer processes the data as per the instructions, and computer provides information” as shown in [10]. |
| Data Provision: | “[…] the process of making data available in an orderly and secure way to users, application developers, and applications that need it […]” as explained in [11]. |
| Data Visualization: | A process to transfer data into understandable visuals. Also described as “[…] Computer-based visualization systems [that] provide visual representations of datasets designed to help people carry out tasks more effectively […]” as Munzner in [12]. |

### **References**

[1] T. Lowdermilk, *User-Centered Design: A Developer’s Guide to Building User-Friendly Applications*. USA: O’Reilly Media, Incorporated, 2013.

[2] G. Giunti, V. Mylonopoulou, and O. R. Romero, “More stamina, a gamified mHealth solution for persons with multiple sclerosis: Research through design,” *JMIR Mhealth Uhealth*, vol. 6, no. 3, p. 4, Mar. 2018, doi: 10.2196/mhealth.9437.

[3] M. Lillemaa, “User-centered design Related papers.”

[4] K. Vredenburg, J.-Y. Mao, P. W. Smith, and T. Carey, “A Survey of User-Centered Design Practice,” 2002.

[5] n.k., *Design Thinking in Higher Education: Interdisciplinary Encounters*. Germany: Springer Singapore, 2020. Accessed: Jan. 19, 2022. [Online]. Available: https://www.google.de/books/edition/Design_Thinking_in_Higher_Education/ZX74DwAAQBAJ?hl=de&gbpv=0

[6] D. R. A. Schallmo, *Jetzt Design Thinking anwenden: In 7 Schritten zu kundenorientierten Produkten und Dienstleistungen*. Germany: Springer Fachmedien Wiesbaden, 2018.

[7] T. Stapelkamp, *Service Design is making Sense – HANDBUCH für Erfolge durch positive Kunden-Erlebnisse*. Leipzig: Institut für User Experience Design, 2018.

[8] L. Løvlie, A. Polaine, and B. Reason, *Service Design: From Insight to Implementation*. USA: Rosenfeld Media, 2013.

[9] M. Stickdorn and J. Schneider, *This is service design thinking: Basics, tools, cases.* . Canada: BIS Publishers, 2012.

[10] J. P. R. Reddy, *Business Data Processing & Computer Applications*. India: APH Publishing Corporation, 2008.

[11] Sybase, “Data Provisioning and Integration.” Accessed: Jan. 19, 2022. [Online]. Available: https://infocenter.sybase.com/help/index.jsp?topic=/com.sybase.stf.df.doc-SWS-2.0.0/projects/datafed/avaki/en/source/c_data_prov_main.html

[12] T. Munzner, “Keynote Speaker Visualization Analysis and Design.”
