# Supplementary material for: Use of Creative Frameworks in Health Care to Solve Data and Information Problems: Scoping Review
Source: JMIR Hum Factors. 2024 Sep 13;11:e55182. doi: 10.2196/55182 (PMC11437220; doi:10.2196/55182)
Supplement: Multimedia Appendix 3 [file humanfactors_v11i1e55182_app3.docx]

**Multimedia Appendix 3**

In the following the overview of the categorization of all 39 papers checked for eligibility. Detailed information concerning the interpreation of data/information problem and creative framework can be found in Multimedia Appendix 3.

Table 1. Overview of included papers during eligibility checking (n=23).

| *Reference* | *Scenario* | *Problem Category* | *Creative Framework* | *Decision* |
| --- | --- | --- | --- | --- |
| Purohit H et al [1] | A | Data Problem  (Data elicitation/data collection) | UCD | Inclusion |
| Burn AM et al [2] | A | Data Problem  (Data elicitation/data collection) | UCD | Inclusion |
| Nuske HJ et al [3] | A | Data Problem (Data elicitation/data collection) | UCD | Inclusion |
| Durski KN et al [4] | A | Data Problem  (Data elicitation/data collection) | Design Thinking | Inclusion |
| Kim MG et al [5] | B | Data Problem (Data elicitation/data collection) | Design Thinking | Inclusion |
| MacHaria P et al [6] | A | Data Problem (Data elicitation/data collection) | UCD | Inclusion |
| Michelle Li AM, Tara Nutley. [7] | A | Data Problem (Data elicitation/data collection) | UCD | Inclusion |
| Karam A et al [8] | A | Data Problem (Data Processing) | UCD | Inclusion |
| Ahufinger S, Herrero [9] | A | Information Problem (Information Provision) | UCD | Inclusion |
| Viderisa NZ et al [10] | A | Information Problem (Information Provision) | UCD | Inclusion |
| Dugstad Wake J et al [11] | A | Information Problem (Information Provision) | Design Science (DSR) | Inclusion |
| Calvo L et al [12] | A | Information Problem (Information Provision) | UCD | Inclusion |
| Davis BM, Dickerson K, Gillmore SC. [13] | A | Information Problem (Information Provision) | UCD | Inclusion |
| Backonja U et al [14] | A | Information Problem (Information Provision) | UCD | Inclusion |
| Desai AD et al [15] | A | Information Problem (Information Provision) | UCD | Inclusion |
| Koopman RJ et al [16] | A | Information Problem (Information Provision) | UCD | Inclusion |
| Almukhalfi H, Goodwin S [17] | A | Information Problem (Information Provision) | UCD, design science research, user-centred design, interface Nielsen’s design principles | Inclusion |
| Kuge J et al [18] | A | Information Problem (Information Provision) | User-centered design process ISO 9241-210, Rapid Contextual Design Framework (for collection and consolidation of data about users and working environment) | Inclusion |
| Crisan A et al [19] | A | Information Problem (Information Provision) | Design Study Methodology | Inclusion |
| Krop P et al [20] | B | Mixed problem (data collection, data processing, information provision) | UCD | Inclusion |
| Tan-McGrory A et al [21] | A | Mixed problem (Data collection, data processing) | HCD | Inclusion |
| Zhou L, Dealmeida D, Parmanto B. [22] | A | Mixed problem (Data collection, data provision) | UCD | Inclusion |
| Rey MF, Freitas CMDS, [23] | A | Mixed problem (Data collection, data processing) | Design Study Methodology | Inclusion |

Table 2. Overview of excluded papers during eligibility checking (n=16).

| *Reference* | *Scenario* | *Problem Category* | *Creative Framework* | *Decision* |
| --- | --- | --- | --- | --- |
| Terrado M, Calvo L, Christel I. [24] | - | - | - | Exclusion.  Duplicate. |
| Wegier P et al. [25] | - | - | - | Exclusion.  Duplicate. |
| Baudet R, Carrau X et al. [26] | - | - | - | Exclusion.  Other Language than English. |
| Anju SS, Sravani B, Madala SR. [27] | - | No data/information problem. | - | Exclusion. |
| Hancock SC et al. [28] | - | No data/information problem. | - | Exclusion. |
| Cunningham M, Cunningham PM [29] | - | No data/information problem. | - | Exclusion. |
| Daniëls NEM, Hochstenbach LMJ, van Bokhoven MA, Beurskens AJHM, Delespaul PAEG. [30] | - | No data/information problem. | - | Exclusion. |
| Ewers A, Gnass I. [31] | - | No data/information problem. | - | Exclusion. |
| Wohofsky L, Krainer D, Schubert P. [32] | - | No data/information problem. |  | Exclusion. |
| Zhang Y, Lugmayr A. [33] | - | No data/information problem. | - | Exclusion. |
| Fareed N et al. [34] | - | - | Insufficient/No description of creative framework. | Exclusion. |
| Emmanuel G, Emanuel AWR, Setyohadi DB. [35] | - | - | Insufficient/No description of creative framework. | Exclusion. |
| Zocha M et al. [36] | - | - | Insufficient/No description of creative framework. | Exclusion. |
| Schubel L et al. [37] | - | - | Insufficient/No description of creative framework. | Exclusion. |
| Theis S et al. [38] | - | - | Insufficient/No description of creative framework. | Exclusion. |
| Zacks JM, Franconeri SL. [39] | - | - | Insufficient/No description of creative framework. | Exclusion. |

**References**

[1] H. Purohit, S. Dubrow, and B. Bannan, “Designing a Multimodal Analytics System to Improve Emergency Response Training,” in *Lecture Notes in Computer Science (including subseries Lecture Notes in Artificial Intelligence and Lecture Notes in Bioinformatics)*, Springer Verlag, 2019, pp. 89–100. doi: 10.1007/978-3-030-21814-0_8.

[2] A. M. Burn, T. J. Ford, J. Stochl, P. B. Jones, J. Perez, and J. K. Anderson, “Developing a Web-Based App to Assess Mental Health Difficulties in Secondary School Pupils: Qualitative User-Centered Design Study,” *JMIR Form Res*, vol. 6, no. 1, Jan. 2022, doi: 10.2196/30565.

[3] H. J. Nuske, J. E. Buck, B. Ramesh, E. M. Becker-Haimes, K. Zentgraf, and D. S. Mandell, “Making Progress Monitoring Easier and More Motivating: Developing a Client Data Collection App Incorporating User-Centered Design and Behavioral Economics Insights,” *Soc Sci*, vol. 11, no. 3, Mar. 2022, doi: 10.3390/socsci11030106.

[4] K. N. Durski *et al.*, “Design thinking during a health emergency: building a national data collection and reporting system,” *BMC Public Health*, vol. 20, no. 1, Dec. 2020, doi: 10.1186/s12889-020-10006-x.

[5] M.-G. Kim, M. Park, Y. S. Choi, K.-H. Kim, N. Lee, and D.-S. Sohn, “Understanding Work Environment of Therapists to Incorporate Robots into Education for Children with Developmental Disorders,” Jeju, Korea: 16th International Conference on Ubiquitous Robots (UR), 2019, p. 791.

[6] P. Macharia *et al.*, “Implementing open data kit integrating barcode-based client identification in HIV testing and linkage to care: A user-centered design,” in *IEEE International Humanitarian TEchnology Conference (HTC)*, 2021. doi: 10.1109/IHTC53077.2021.9698914.

[7] M. Li, A. Makulec, and T. Nutley, *Applying User-Centered Design to Data Use Challenges: What We Learned Applying User-Centered Design to Data Use Challenges*. 2017. [Online]. Available: www.measureevaluation.org

[8] A. Karam, T. M. Illemann, K. H. Reinau, G. Vuk, and C. O. Hansen, “Towards deriving freight traffic measures from truck movement data for state road planning: a proposed system framework,” *ISPRS Int J Geoinf*, vol. 9, no. 10, Oct. 2020, doi: 10.3390/ijgi9100606.

[9] S. Ahufinger and P. Herrero, “3D brain connectivity visualization for medical systems,” in *Proceedings - IEEE Symposium on Computer-Based Medical Systems*, Institute of Electrical and Electronics Engineers Inc., Jun. 2019, pp. 9–13. doi: 10.1109/CBMS.2019.00013.

[10] N. Z. Viderisa, H. B. Santoso, and R. Y. K. Isal, *Designing the Prototype of Personalized Push Notifications on E-Commerce Application with the User-Centered Design Method*. 2019.

[11] J. Dugstad Wake, F. Rabbi, Y. Inal, and T. Nordgreen, “User-centred design of clinical dashboards for guided iCBT,” *Innov Syst Softw Eng*, 2022, doi: 10.1007/s11334-022-00454-6.

[12] L. Calvo, I. Christel, M. Terrado, F. Cucchietti, and M. Pérez-Montoro, “Users’ Cognitive Load: A Key Aspect to Successfully Communicate Visual Climate Information,” *Bull Am Meteorol Soc*, vol. 103, no. 1, pp. E1–E16, Jan. 2022, doi: 10.1175/BAMS-D-20-0166.1.

[13] B. M. Davis, K. Dickerson, and S. C. Gillmore, “User centered design strategies for improving visualization of sensor data in rotorcraft cockpit displays for degraded visual environment operations,” in *Advances in Intelligent Systems and Computing*, Springer Verlag, 2020, pp. 131–141. doi: 10.1007/978-3-030-19135-1_13.

[14] U. Backonja, S. C. Haynes, and K. K. Kim, “Data visualizations to support health practitioners’ provision of personalized care for patients with cancer and multiple chronic conditions: User-centered design study,” *JMIR Hum Factors*, vol. 5, no. 4, Oct. 2018, doi: 10.2196/11826.

[15] A. D. Desai *et al.*, “Caregiver and Health Care Provider Perspectives on Cloud-Based Shared Care Plans for Children With Medical Complexity,” 2018, doi: 10.1542/hpeds.2017-0242.

[16] R. J. Koopman *et al.*, “Home blood pressure data visualization for the management of hypertension: Designing for patient and physician information needs,” *BMC Med Inform Decis Mak*, vol. 20, no. 1, Aug. 2020, doi: 10.1186/s12911-020-01194-y.

[17] H. Almukhalfi and S. Goodwin, “Visually Communicating Microgrid Complexity,” in *ACM International Conference Proceeding Series*, Association for Computing Machinery, Nov. 2021, pp. 614–619. doi: 10.1145/3487664.3487749.

[18] J. Kuge, T. Grundgeiger, P. Schlosser, P. Sanderson, and O. Happel, “Design and Evaluation of a Head-Worn Display Application for Multi-Patient Monitoring,” in *DIS 2021 - Proceedings of the 2021 ACM Designing Interactive Systems Conference: Nowhere and Everywhere*, Association for Computing Machinery, Inc, Jun. 2021, pp. 879–890. doi: 10.1145/3461778.3462011.

[19] A. Crisan, G. McKee, T. Munzner, and J. L. Gardy, “Evidence-based design and evaluation of a whole genome sequencing clinical report for the reference microbiology laboratory,” *PeerJ*, vol. 2018, no. 1, 2018, doi: 10.7717/peerj.4218.

[20] P. Krop, S. Straka, M. Ullrich, M. Ertl, and M. E. Latoschik, “IT-Supported request management for clinical radiology: Analyzing Requirements through Contextual Interviews,” in *ACM International Conference Proceeding Series*, Association for Computing Machinery, Sep. 2021, pp. 120–124. doi: 10.1145/3473856.3473992.

[21] A. Tan-McGrory *et al.*, “A patient and family data domain collection framework for identifying disparities in pediatrics: Results from the pediatric health equity collaborative,” *BMC Pediatr*, vol. 18, no. 1, Jan. 2018, doi: 10.1186/s12887-018-0993-2.

[22] L. Zhou, D. Dealmeida, and B. Parmanto, “Applying a user-centered approach to building a mobile personal health record app: Development and usability study,” *JMIR Mhealth Uhealth*, vol. 7, no. 7, Jul. 2019, doi: 10.2196/13194.

[23] M. F. Rey and C. M. D. S. Freitas, “Interactive Visualizations to Support Randomized Clinical Trial Monitoring.”

[24] M. Terrado, L. Calvo, and I. Christel, “Towards more effective visualisations in climate services: good practices and recommendations,” *Climatic Change*, vol. 172, no. 1–2. Springer Science and Business Media B.V., May 01, 2022. doi: 10.1007/s10584-022-03365-4.

[25] P. Wegier *et al.*, “Home blood pressure data visualization for the management of hypertension: using human factors and design principles,” *BMC Med Inform Decis Mak*, vol. 21, no. 1, Dec. 2021, doi: 10.1186/s12911-021-01598-4.

[26] R. Baudet *et al.*, “Contribution of clinical data to the design of assistive systems,” in *IHM 2019 - Annexes des Actes de la 31e Conference Francophone sur l’Interaction Homme-Machine*, Association for Computing Machinery, Inc, Dec. 2019. doi: 10.1145/3366551.3370351.

[27] S. Sumi Anju, B. Sravani, and S. Rao Madala, “Publicly Verifiable Vibrant Digital Medical Information Systems,” 2021.

[28] S. C. Hancock *et al.*, “Introducing the combined atlas framework for large-scale web-based data visualization: The GloNAF atlas of plant invasion,” *Methods Ecol Evol*, vol. 13, no. 5, pp. 1073–1081, May 2022, doi: 10.1111/2041-210X.13820.

[29] M. Cunningham and P. M. Cunningham, “mHealth4Afrika Pilot Validation in Healthcare Facilities in Ethiopia, Kenya and Malawi,” in *IEEE Global Humanitarian Technology Conference (GHTC)*, 2019.

[30] N. E. M. Daniëls, L. M. J. Hochstenbach, M. A. van Bokhoven, A. J. H. M. Beurskens, and P. A. E. G. Delespaul, “Implementing Experience Sampling Technology for Functional Analysis in Family Medicine – A Design Thinking Approach,” *Front Psychol*, vol. 10, Dec. 2019, doi: 10.3389/fpsyg.2019.02782.

[31] A. Ewers and I. Gnass, “painApp—mobile pain monitoring in the home care setting,” *Schmerz*, vol. 32, no. 5. Springer Verlag, pp. 348–355, Oct. 01, 2018. doi: 10.1007/s00482-018-0313-7.

[32] L. Wohofsky, D. Krainer, and P. Schubert, “Telerehabilitation platform REHA2030: Visualization of training statistics for patient and therapist,” in *ACM International Conference Proceeding Series*, Association for Computing Machinery, Jun. 2021, pp. 252–253. doi: 10.1145/3453892.3461005.

[33] Y. Zhang and A. Lugmayr, “Designing a User-Centered Interactive Data-Storytelling Framework,” *Association for Computing Machinery*, 2019, doi: https://doi.org/10.1145/3369467.3369507.

[34] N. Fareed, C. M. Swoboda, P. Jonnalagadda, T. Griesenbrock, H. R. Gureddygari, and A. Aldrich, “Visualizing Opportunity Index Data Using a Dashboard Application: A Tool to Communicate Infant Mortality-Based Area Deprivation Index Information,” *Appl Clin Inform*, vol. 11, no. 4, pp. 515–527, Aug. 2020, doi: 10.1055/s-0040-1714249.

[35] G. Emmanuel, A. W. R. Emanuel, and D. B. Setyohadi, “Design of mobile application for community health workers: A case study in Rwanda,” *International Journal of Interactive Mobile Technologies*, vol. 14, no. 11, pp. 271–279, 2020, doi: 10.3991/ijim.v14i11.13307.

[36] M. Zocha *et al.*, “Adaption of the OMOP CDM for rare diseases,” in *Public Health and Informatics: Proceedings of MIE 2021*, IOS Press, 2021, pp. 138–142. doi: 10.3233/SHTI210136.

[37] L. Schubel *et al.*, “Informatics and interaction: Applying human factors principles to optimize the design of clinical decision support for sepsis,” *Health Informatics J*, vol. 26, no. 1, pp. 642–651, Mar. 2020, doi: 10.1177/1460458219839623.

[38] S. Theis, P. W. Victor Rasche, C. Bröhl, M. Wille, and A. Mertens, “Task-data taxonomy for health data visualizations: Web-based survey with experts and older adults,” in *JMIR Medical Informatics*, Jul. 2018. doi: 10.2196/medinform.9394.

[39] J. M. Zacks and S. L. Franconeri, “Designing Graphs for Decision-Makers,” *Policy Insights Behav Brain Sci*, vol. 7, no. 1, pp. 52–63, Mar. 2020, doi: 10.1177/2372732219893712.
