# Supplementary material for: Use of Creative Frameworks in Health Care to Solve Data and Information Problems: Scoping Review
Source: JMIR Hum Factors. 2024 Sep 13;11:e55182. doi: 10.2196/55182 (PMC11437220; doi:10.2196/55182)
Supplement: Multimedia Appendix 4 [file humanfactors_v11i1e55182_app4.docx]

**Multimedia Appendix 4**

In the following six examples of included (n=2) and excluded papers (n=4) are shown.

###### **Example 1 included: Design and Evaluation of a Head-Worn Display Application for Multi-Patient Monitoring [1].**

*Abstract:* “Supervising anesthesiologists oversee junior anesthesiologists in several operating rooms to instruct and provide advice, and hence ensure high patient safety. Supervisors benefit from good situation awareness but accessing information about multiple patients can be challenging. In a user-centered design process, we have developed an interface concept for a head-worn display (HWD) to support multi-patient monitoring. […]”.

*Problem Statement:* The problem revolves around supervising anesthesiologists in maintaining good situation awareness while overseeing multiple patients in different operating rooms. Accessing information about multiple patients is identified as a challenge within this context.

*Information Problem:* The problem described can be categorized as an information problem. That is because the primary concern is with the accessibility and presentation of information (i.e., patient data) rather than the processing or storage of data itself. We therefore conclude an information problem.

*Application of Creative Framework:* A creative framework, specifically a user-centered design process, was applied to address the identified challenge. The development of an interface concept for a head-worn display (HWD) is the creative solution proposed in response to the problem statement. The user-centered design process emphasizes understanding the needs and preferences of the end-users (supervising anesthesiologists) to create an interface that supports multi-patient monitoring effectively.

###### Conclusion: Data/information problem: yes, creative framework: yes.

###### **Example 2 included: Implementing open data kit integrating barcode-based client identification in HIV testing and linkage to care: A user-centered design [2].**

*Abstract:* “mHealth has improved HIV interventions worldwide. Electronic data collection has made it possible to monitor HIV in near real-time. However, the pandemic in low and middle-income countries seem to be evolving in a manner leading many to believe the Sustainable Development Goal 3.3 goal of eliminating HIV by 2030 may not be achieved. The use of paper-based health records and lack of universal client unique identification may be contributing to this trend. We aimed to evaluate how data collection integrating barcode identifiers could improve HIV care. A user-centred design (UCD) approach guided the customization of open data kit integrating barcodes. […]”

*Problem Statement:* Two specific data problems are highlighted: the use of paper-based health records and the lack of universal client-unique identification.

*Data Problem:* The described problem can be categorized as a data and an information problem. The use of paper-based health records suggests a data problem, as it implies challenges related to data collection, storage, and accessibility. Additionally, the lack of unique, universal client identification points to an information problem, as it highlights issues with accurately identifying and tracking individuals within the healthcare system.

*Application of Creative Framework:* A creative framework, specifically a user-centered design (UCD) approach, was applied to address the identified challenges. The customization of an open data kit integrating barcodes for data collection in HIV care is the creative solution proposed in response to the problem statement. The UCD approach guided the design process to ensure that the data collection system met the needs and preferences of end-users (healthcare providers and HIV patients) effectively.

###### Conclusion: Data/information problem: yes, creative framework: yes.

###### **Example 3 excluded: Publicly Verifiable Vibrant Digital Medical Information Systems [3].**

*Abstract:* “As data processing advances, decentralized media has been widely recognized for its ability to store large amounts of data. By comparing a revisited content to a dispersed repository, a cloud provider may verify the document's integrity without having to retrieve it. A reconsidered examining strategy is offered to lead the customer to reconsider the significant assessing task to third sector inspector, taking into consideration the important computing cost brought up by the checking process (TPA). TPA may be deterred by the primary revisited evaluating strategy, but the second plan gives the harmful organization the right of inspection over the readdressed data of users, which poses a significant risk to patient privacy. Human Emphasis for reconsidered inspection is presented in this work, which emphasizes that the service user can be overwhelmed by her own data. Based on user-centered design, our suggested methodology not only prevents patient's data from leaking to TPA without depending on cryptographic algorithms, but can also avoid the use of additional free unpredictable supply that is impossible to fulfill on a daily basis. Also, we start to make our approach work with continuous changes. Our recommended scheme is both verifiably safe and essentially productive, as shown by the privacy analysis and test evaluations.[…]”

*Problem Statement:* The problem statement is not totally clear. Both reviewers decided independently to not include this paper due to a lack of problem statement and data/information problem. It could be concluded, that the problem points to document integrity, but the abstract is not written from a problem perspective e.g., Over the last years a total of X patient data breaches happened. Stakeholders wish for more security etc.

*Data or Information Problem:* None or not clear enough.

*Application of Creative Framework:* A creative framework (UCD) is applied.

*Conclusion:* Data/information problem: no, creative framework: yes.

###### **Example 4 excluded: Introducing the combined atlas framework for large-scale web-based data visualization: The GloNAF atlas of plant invasion** [4]

*Abstract:* “Large-scale biodiversity data, for example, on species distribution and richness information, are being mobilized and becoming available at an increasing rate. Interactive web applications like atlases have been developed to visualize available datasets and make them accessible to a wider audience. Web mapping tools are changing rapidly, and different underlying concepts have been developed to visualize datasets at a high cartographic standard. Here, we introduce the Combined Atlas Framework for the development of interactive web atlases for ecological data visualization. We combine two existing approaches: the five stages of the user-centred design approach for web mapping applications and the three U approach for interface success. Subsequently, we illustrate the use of this framework by developing the Atlas of Plant Invasions based on the Global Naturalized Alien Flora (GloNAF) database. This case study illustrates how the newly developed Combined Atlas Framework with a user-centred design philosophy can generate measurable success through communication with the target user group, iterative prototyping and competitive analysis of other existing web mapping approaches. The framework is useful in creating an atlas that employs user feedback to determine usability and utility features within an interactive atlas system. Finally, this framework will enable a better-informed development process of future visualization and dissemination of biodiversity data through web mapping applications and interactive atlases. […]”

*Problem Statement:* The problem statement is not totally clear. Both reviewers decided independently to not include this paper due to a lack of problem statement and data/information problem. It could be concluded, that due to the increasing rate of diversity of data and its distribution new visualization tools are needed to view these datasets but it is not totally clear.

*Data or Information Problem:* None or not clear enough.

*Application of Creative Framework:* A creative framework (UCD) and the three U approach is applied.

###### Conclusion: Data/information problem: no, creative framework: yes.

###### **Example 5 excluded: Task-data taxonomy for health data visualizations: Web-based survey with experts and older adults** [5]**.**

*Abstract:* “Increasingly, eHealth involves health data visualizations to enable users to better understand their health situation. Selecting efficient and ergonomic visualizations requires knowledge about the task that the user wants to carry out and the type of data to be displayed. Taxonomies of abstract tasks and data types bundle this knowledge in a general manner. Task-data taxonomies exist for visualization tasks and data. They also exist for eHealth tasks. However, there is currently no joint task taxonomy available for health data visualizations incorporating the perspective of the prospective users. One of the most prominent prospective user groups of eHealth are older adults, but their perspective is rarely considered when constructing tasks lists. Objective: The aim of this study was to construct a task-data taxonomy for health data visualizations based on the opinion of older adults as prospective users of eHealth systems. eHealth experts served as a control group against the bias of lacking background knowledge. The resulting taxonomy would then be used as an orientation in system requirement analysis and empirical evaluation and to facilitate a common understanding and language in eHealth data visualization. Methods: Answers from 98 participants (51 older adults and 47 eHealth experts) given in an online survey were quantitatively analyzed, compared between groups, and synthesized into a task-data taxonomy for health data visualizations. Results: Consultation, diagnosis, mentoring, and monitoring were confirmed as relevant abstract tasks in eHealth. Experts and older adults disagreed on the importance of mentoring (χ24=14.1, P=.002) and monitoring (χ24=22.1, P&lt;.001). The answers to the open questions validated the findings from the closed questions and added therapy, communication, cooperation, and quality management to the aforementioned tasks. Here, group differences in normalized code counts were identified for “monitoring” between the expert group (mean 0.18, SD 0.23) and the group of older adults (mean 0.08, SD 0.15; t96=2431, P=.02). Time-dependent data was most relevant across all eHealth tasks. Finally, visualization tasks and data types were assigned to eHealth tasks by both experimental groups. Conclusions: We empirically developed a task-data taxonomy for health data visualizations with prospective users. This provides a general framework for theoretical concession and for the prioritization of user-centered system design and evaluation. At the same time, the functionality dimension of the taxonomy for telemedicine—chosen as the basis for the construction of present taxonomy—was confirmed. […]”

*Problem Statement:* The problem statement focuses on the absence of a comprehensive task-data taxonomy for health data visualizations that incorporates the perspective of older adults as prospective users of eHealth systems. This lack of a joint taxonomy limits the ability to select efficient and ergonomic visualizations tailored to the needs of older adult users, thereby hindering effective health data communication and understanding.

*Data or Information Problem:* The problem described primarily points to an information problem. The challenge revolves around organizing and categorizing information (i.e., health data visualizations) to facilitate effective communication and understanding among older adult users of eHealth systems.

*Application of Creative Framework:* None or at least none mentioned.

###### Conclusion: Data/information problem: yes, creative framework: no.

###### **Example 6 excluded: Designing Graphs for Decision-Makers** [6]**.**

*Abstract:* “Data graphics can be a powerful aid to decision-making—if they are designed to mesh well with human vision and understanding. Perceiving data values can be more precise for some graphical types, such as a scatterplot, and less precise for others, such as a heatmap. The eye can extract some types of statistics from large arrays in an eyeblink, as quickly as recognizing an object or face. But perceiving some patterns in visualized numbers—particularly comparisons within a dataset—is slow and effortful, unfolding over a series of operations that are guided by attention and previous experience. Effective data graphics map important messages onto visual patterns that are easily extracted, likely to be attended, and as consistent as possible with the audience’s previous experience. User-centered design methods, which rely on iteration and experimentation to improve a design, are critical tools for creating effective data visualizations. […]”

*Problem Statement:* The described problem focuses on the visualization of data graphics. The authors point out that the perception of some patterns visualized in numbers is slow and effortful.

*Data or Information Problem:* The problem described primarily points to an information problem.

*Application of Creative Framework:* The authors mention User-Centered Design methods in general but not if they applied them to solve their problem.

###### Conclusion: Data/information problem: yes, creative framework: no.
